# Nutrition in Inflammatory Bowel Disease: Strategies to Improve Prognosis and New Therapeutic Approaches

**DOI:** 10.3390/diseases13050139

**Published:** 2025-05-01

**Authors:** Nallely Bueno-Hernández, Jesús K. Yamamoto-Furusho, Viridiana Montsserrat Mendoza-Martínez

**Affiliations:** 1 Proteomics and Metabolomics Laboratory, Research Division, General Hospital of Mexico “Dr. Eduardo Liceaga”, Mexico City 06720, Mexico; viridiana_2909@hotmail.com; 2 Inflammatory Bowel Disease Clinic, Department of Gastroenterology, Instituto Nacional de Ciencias Médicas y Nutrición Salvador Zubirán, Mexico City 14080, Mexico; kazuofurusho@hotmail.com

**Keywords:** inflammatory bowel disease, Crohn’s disease, ulcerative colitis, nutritional assessment, enteral nutrition, parenteral nutrition, dietary therapy

## Abstract

Inflammatory Bowel Disease (IBD), encompassing Crohn’s disease (CD) and ulcerative colitis (UC), is a chronic inflammatory condition of the gastrointestinal tract that significantly impacts nutritional status. Malnutrition is a frequent complication, resulting from reduced nutrient intake, malabsorption, and increased metabolic demands due to chronic inflammation. A comprehensive nutritional assessment encompassing anthropometric, biochemical, and dietary evaluations is crucial for informing personalized interventions. Several nutritional approaches have been explored to modulate inflammation and the gut microbiota, yielding promising results. The Mediterranean, anti-inflammatory, and low-FODMAP diets have shown potential benefits in symptom control. In contrast, diets high in ultra-processed foods and saturated fats are associated with worsened disease activity. Additionally, stool consistency, assessed using the Bristol Stool Scale, serves as a practical indicator for dietary adjustments, helping to regulate fiber intake and hydration strategies. When dietary modifications alone are insufficient, nutritional support becomes a critical component of IBD management. Enteral nutrition (EN) is preferred whenever possible because it maintains gut integrity and modulates immune responses. It has demonstrated efficacy in reducing postoperative complications and improving disease control. In cases where EN is not feasible, such as in intestinal obstruction, severe malabsorption, or high-output fistulas, parenteral nutrition (PN) is required. The choice between peripheral and central administration depends on treatment duration and osmolarity considerations. Despite growing evidence supporting nutritional interventions, further research is needed to establish standardized guidelines that optimize dietary and nutritional support strategies in managing IBD.

## 1. Introduction

Inflammatory Bowel Disease (IBD) mainly consists of two diseases: Crohn’s disease (CD) and ulcerative colitis (UC). The assessment of these diseases requires a multidisciplinary approach that considers the clinical presentation of the disease, the patient’s nutritional status, and dietary habits. When taking a nutritional history, it is essential to assess body composition, biochemical parameters, physical activity, and clinical manifestations of nutritional status [1]. Nutritional care in IBD patients is crucial, focusing on preventing malnutrition, specific micronutrient deficiencies, and adverse changes in body composition. Changes in nutritional status are more common during the active phases of the disease; therefore, timely screening, diagnosis, and follow-up are essential [2,3].

This review aims to provide a comprehensive and clinically oriented synthesis of the relationship between IBD and nutritional status, focusing on aspects often underrepresented in the previous literature. It explores the efficacy of dietary interventions, the potential risks of non-recommended diets, and the role of nutritional support in improving clinical outcomes. This work seeks to offer a more practical and updated perspective to support clinical decision-making in the nutritional care of IBD patients.

## 2. Impact of Intestinal Inflammation on Nutritional Status

Intestinal inflammation impairs nutrient absorption through several interrelated mechanisms. Firstly, inflammation damages the intestinal mucosa, including the villi and microvilli, which are crucial in maximizing the surface area for nutrient uptake. The resulting structural loss significantly reduces the absorption efficiency of macronutrients and micronutrients such as carbohydrates, proteins, lipids, vitamins, and minerals. In addition, inflammation disrupts the tight junctions between epithelial cells, leading to increased intestinal permeability, commonly called “leaky gut.” This allows microbial products and toxins to be translocated into the systemic circulation, exacerbating the immune response and further impairing nutrient transport [4,5].

Moreover, inflammation induces dysbiosis, characterized by alterations in the composition and function of the gut microbiota, decreasing the abundance of Firmicutes, the *Bifidobacterium* genus, and *Faecalibacterium prausnitzii*. [6,7]. This imbalance compromises the microbial fermentation of dietary fibers, the production of short-chain fatty acids, and the synthesis of essential vitamins, such as vitamin K and several B-complex vitamins. Inflammatory cytokines, including tumor necrosis factor-alpha (TNF-α) and interleukin-1 beta (IL-1β), also downregulate the expression and function of nutrient transporters, such as those for glucose (e.g., SGLT1 and GLUT2), amino acids, and fatty acids [4,5].

Finally, inflammation may accelerate intestinal transit and promote osmotic or secretory diarrhea, reducing the time available for nutrient absorption and contributing to secondary malabsorption. These combined effects can significantly compromise nutritional status, especially in individuals with IBD [4,5,8]. Specifically, in CD patients, the absorption of vitamin B12, fats, fat-soluble vitamins (A, D, E, K), calcium, iron, and proteins is affected due to the involvement of different parts of the gastrointestinal tract, including the ileum and colon. In UC, malabsorption is less common, but vitamin D, calcium, iron, and proteins can be compromised due to chronic inflammation and blood loss [9,10,11]. These mechanisms underscore the importance of addressing intestinal inflammation to maintain optimal nutrient absorption and nutritional complications.

## 3. Nutritional Complications in IBD Patients

Malnutrition is an essential problem in patients with IBD, with a prevalence of 20–85% [12]. According to the literature, the frequency of protein–energy malnutrition in patients with active IBD is 75%. It is an essential problem because it is related to an increased risk of infections, poor prognosis in patients, and post-surgery complications [13]. These complications can have a direct impact on the patient’s prognosis and increase the risk of morbidity and mortality [14].

Patients may be partially or utterly deficient in various nutrients. According to ESPEN, there is a higher risk of micronutrient deficiencies, including vitamins such as vitamin A, folic acid, vitamin B6, vitamin B12, vitamin D, and vitamin K, and minerals such as iron, zinc, calcium, and selenium. These deficiencies are associated with insufficiency of the alimentary tract caused by diarrhea, malabsorption, intestinal failure, and inadequate dietary consumption secondary to anorexia accompanying disease activity [5,13,15]. In conjunction with acute intestinal inflammation, there is an increase in basal energy, protein catabolism, anemia, osteoporosis, thrombophilia, poor wound healing, and even an exacerbation of chronic inflammation and carcinogenesis [16]. Recent studies have shown a higher prevalence of malnutrition in CD patients and a greater vulnerability to folate and vitamin B12 deficiencies due to the ileocecal location and sulfasalazine administration compared to UC patients [17]. Therefore, assessing the nutritional status of patients with IBD is crucial as it is essential in disease management and overall health outcomes.

## 4. Nutritional Assessment in IBD

**Figure 1:**
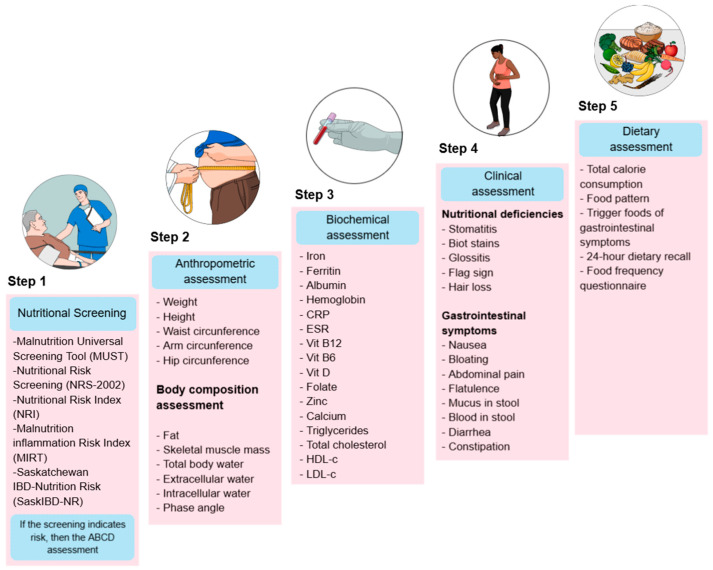
Nutritional assessment in patients with IBD. The image outlines a five-step process for nutritional evaluation. CRP: C-Reactive Protein, ESR: Erythrocyte Sedimentation Rate, Vit B12: Vitamin B12, Vit B6: Vitamin B6, Vit D: Vitamin D, HDL-c: High-Density Lipoprotein Cholesterol, LDL-c: Low-Density Lipoprotein Cholesterol.

Nutritional treatment is a comprehensive approach to patient care that aims to identify dietary risks at different stages of the disease, prevent deficiencies, and improve treatment response. To achieve this, nutritional assessment is commonly structured into five key components (Figure 1).

**Step 1 Screening:** Nutritional screening tools (NSTs) are frequently used in clinical practice to identify patients at risk of malnutrition because they are rapid evaluations that any medical team member can complete. In contrast, nutrition assessment tools (NATs) are usually more detailed and require specialized resources [18]. The ESPEN guidelines for clinical nutrition in IBD state that patients are mainly at risk of malnutrition, and it is recommended that they be screened for malnutrition at the time of diagnosis and then regularly during follow-up [19]. Nutritional Risk Screening 2002 (NRS-2002), Malnutrition Universal Screening Tool (MUST), Malnutrition Screening Tool (MST), Nutritional Risk Index (NRI), Global Leadership Initiative on Malnutrition (GLIM), index based on laboratory parameters as controlling nutritional status (CONUT) and onodera prognostic dietary index (OPNI), as well as IBD specific tools, such as the Malnutrition Inflammation Risk Tool (MIRT) and the Saskatchewan IBD–Nutrition Risk (SaskIBD-NR), are the most frequently used [20,21,22,23]. A comprehensive nutritional assessment begins with a nutritional screening to identify patients at risk of malnutrition, enabling timely intervention.

**Step 2 Body metrics:** Anthropometric and body composition studies are crucial for monitoring changes in muscle mass and adipose tissue, as IBD often leads to muscle wasting and fat loss due to chronic inflammation and inadequate nutritional intake; recently, phase angle (PhA) has been proposed as a marker to assess cellular integrity and nutritional status in UC patients [11,24].

**Step 3 Biochemical assessment:** Biochemical testing helps identify macronutrient and micronutrient status and monitor inflammatory markers and effects of medication that can exacerbate nutritional problems because some micronutrients are influenced by disease activity, consequently producing malabsorption syndrome, dehydration, anemia, osteoporosis, and in specific cases, hypercholesterolemia in part due to improved management of IBD and the obesity epidemic [25].

**Step 4: Clinical assessment:** Gastrointestinal symptoms and clinical manifestations of nutritional deficiencies are evaluated to assess the impact of inflammation, diarrhea, abdominal pain, and anorexia on nutritional status. Identifying bowel symptoms in inactive IBD is vital to avoid unnecessary and potentially harmful treatment strategies [11,25].

**Step 5 Dietary assessment**: Analyze the patient’s food intake and make the necessary changes because many patients with IBD alter their diet to control their symptoms, whether during periods of active disease or remission. Indeed, these self-induced dietary restrictions may harm patients’ nutritional status. Therefore, supervision by a dietitian may be required to improve nutrient intake [9,10,11,25].

Together, these components provide a holistic approach to UC management that helps prevent malnutrition, customizes nutritional interventions, improves patient outcomes, and initiates nutritional treatment [19,26,27,28].

## 5. Diet Therapies in IBD

Numerous studies have shown that diet content is among the most significant environmental factors influencing the activity of IBD, affecting both the disease’s etiology and progression. This is because the intestinal microbiota, immune system, and epithelial barrier function are regulated by metabolites, vitamins, and minerals derived from food. Therefore, dietary therapies have gained attention as a potential means to induce remission and prevent disease progression in patients with IBD. Many studies evaluate dietary therapies to induce remission in patients and to avoid disease progression.

### 5.1. Mediterranean Diet

The Mediterranean diet is linked to a lower risk of cardiovascular diseases and chronic conditions [3]. This diet emphasizes unprocessed, anti-inflammatory foods, promoting a diet rich in microbiota-accessible carbohydrates, lean protein, and omega-3 fatty acids. Specifically in IBD, the diet stimulates the production of short-chain fatty acids (SCFAs), which help alleviate gastrointestinal symptoms and regulate inflammation and immune responses [29]. Additionally, in patients with active diseases, the Mediterranean diet has been shown to reduce disease activity and markers of inflammation, such as fecal calprotectin and C-reactive protein (CRP). It may also improve liver steatosis in those with concurrent metabolic dysfunction-associated steatotic liver disease (MASLD) [29,30]. This diet has been shown to offer several benefits to patients in remission, such as reducing inflammation and improving overall health outcomes. The best practice guidelines recommend encouraging all patients with IBD to adopt the MD approach, which can improve gut health and manage inflammation [31]. However, research is needed to understand the specific benefits of this approach in UC and CD patients.

### 5.2. Dairy-Free Diet

On the other hand, the use of dairy products in IBD is a topic of controversy, with varying information available. Some dairy foods have been found to contain sulfur-containing products that are harmful to colon cells, as they increase the concentration of hydrogen sulfide in the intestine [32]. Also, lactose-containing products can cause intolerance, leading to clinical symptoms due to a lactase deficiency at the brush border of the small intestine. This deficiency prevents proper absorption, increases osmolarity, and triggers bacterial fermentation, producing hydrogen, carbon dioxide, and methane, which leads to increased abdominal inflammation and diarrhea. Although the lactose content of all dairy products is similar, it has been confirmed that fermented dairy products are better tolerated [3]. However, the use of a dairy-free diet in IBD remains controversial, as it has not been reported that IBD patients are more lactose intolerant compared to the general population [33]. Yilmaz et al. demonstrated that consuming 400 mL of kefir once daily for 4 weeks helps regulate gut microbiota, improves abdominal pain, intestinal inflammation, stool frequency, stool consistency, and quality of life in patients. Specifically, CD patients significantly decreased the erythrocyte sedimentation rate and CRP, while hemoglobin levels increased after 2 weeks. A dairy-free diet should be cautiously recommended, as it can lead to vitamin D and calcium deficiencies, contributing to bone demineralization in IBD patients [3,34].

### 5.3. Anti-Inflammatory Diet

In 2014, researchers investigated the impact of an anti-inflammatory diet tailored for individuals with IBD (IBD-AID). This diet focused on five main aspects: reducing monosaccharides and disaccharides, increasing consumption of prebiotics and probiotics, incorporating healthy fats like monounsaturated and polyunsaturated fats, identifying nutritional deficiencies, and recognizing food intolerances. The diet was also adjusted for texture based on the patient’s symptoms if necessary. After three months, patients with varying levels of IBD improved their condition, as measured by the Harvey–Bradshaw Index and the modified Truelove and Witts severity index [35]. The Groningen diet (GrAID) is considered one of the most promising anti-inflammatory diets. It consists of three key strategies: boosting the intake of foods with anti-inflammatory effects to support beneficial gut bacteria; reducing the consumption of foods linked to pro-inflammatory responses by limiting sodium, sugar, red meats, processed meats, additives, alcohol; and ensuring the diet is well-balanced so patients can identify food intolerances through experimentation [36]. Anti-inflammatory diets show promise in managing IBD, but more scientific evidence is needed to confirm their effectiveness.

### 5.4. High-Fiber Diet

Dietary fibers can be broadly classified into two main types: insoluble fibers, such as cellulose, hemicellulose, and lignin, and soluble fibers, such as pectins, gums, and mucilages, which form a viscous gel-like consistency when mixed with water. A systematic review assessed the impact of various fiber types on patients with IBD. Although the evidence is limited, the findings suggest that fibers like inulin and barley fiber may hold promise in reducing IBD activity scores [37].

Recent studies have shown that fiber consumption has a prebiotic effect, as the metabolites generated by microbiota, such as butyrate, serve as protective factors against IBD activity and decrease the risk of colon cancer. However, controversy exists, as recent studies suggest potential confounding factors, such as the presence of gluten and Fermentable Oligo-, Di-, Monosaccharides, and Polyols (FODMAPs), as well as other dietary components, that could negatively impact patient symptoms [29,32].

In contrast, vegetarian diets have been shown to minimize the consumption of processed foods, animal products, and saturated fats, while promoting a higher intake of vegetables, fruits, legumes, nuts, whole grains, and soy-rich foods, which could have anti-inflammatory effects compared to a standard diet. The mechanism by which these benefits are provided has not been clearly described, and long-term effects with a large sample size have not been demonstrated in UC and CD patients [2,38]. Specifically, a cohort study found over 6 months, daily consumption of 24 g of fiber in patients with CD was associated with a 40% lower risk of disease than in those who consumed less than 10 g of fiber and avoided fiber-rich foods. No associations were found between fiber intake and flare-ups in UC patients [39].

Fiber intake is recommended to primarily consist of soluble fiber, which dissolves in water to form a gel. This gel helps reduce intestinal motility and improve the consistency of stools. Food rich in soluble fiber includes fruits such as apples and bananas, cereals like oats and rice, and legumes like beans and lentils [40]. Although data are still limited, existing research does not support avoiding fiber in remission states.

### 5.5. Low-FODMAP Diet

The low-FODMAP diet (LFD) is a diet that eliminates fermentable foods that trigger symptoms in individuals who are sensitive to them. These foods are poorly absorbed in the small intestine, remain in the intestinal lumen, exert an osmotic effect that draws water into the lumen, and are fermented by colonic bacteria in the colon, producing gases and organic acids. The use of the LFD has been suggested as a potential therapeutic aid for IBD patients, as it has yielded good results, particularly in patients with UC, as well as other bowel disorders such as irritable bowel syndrome (IBS) [28]. The implementation of this diet consists of two phases: a restriction phase, during which all foods high in FODMAPs are avoided for 6 to 8 weeks, and an exposure phase, in which these foods are reintroduced and their tolerance is assessed both qualitatively and quantitatively in each patient [15,41,42,43]. In IBD patients, LFD has been associated with improvements in fecal markers of intestinal inflammation, including fecal calprotectin, as well as enhanced quality of life and reduced disease activity. The most significant benefit has been observed in patients with an overlap of symptoms of IBS, some functional digestive disorders, and early stages of IBD, with substantial clinical improvement in all symptoms except constipation [42,43,44]. On the other hand, the benefits of this diet have been attributed to a reduction in gluten consumption, as low gluten consumption has been associated with fewer symptoms, including bloating, abdominal pain, diarrhea, and nausea, even in patients without celiac disease [45]. Although LFD has shown promising results in IBD patients, further studies are needed to assess its long-term effects on nutritional status.

### 5.6. Dietary Management Based on Bowel Function and Disease Activity in IBD

Dieting plays a crucial role in the gastrointestinal health of patients with IBD. As classified by the Bristol Stool Scale, stool consistency reflects bowel function and can be influenced by dietary habits, hydration, and disease activity. Nutritional interventions can help manage stool consistency, prevent complications, and improve overall gastrointestinal function [46].

During the active phase of Inflammatory Bowel Disease, especially in symptoms such as diarrhea, abdominal pain, or stricture, a low-residue diet is recommended, with well-cooked foods that are low in insoluble fiber and free of irritants. It is preferable to prioritize bland preparations such as rice, mashed potatoes, cooked carrots, boiled chicken, and stewed fruit. These options help reduce fecal volume, minimize intestinal stimulation, and improve digestive tolerance. In severe cases, enteral nutrition may be considered a therapeutic strategy [11,37].

Particularly in active CD or stenosing complications, liquid formulas effectively induce remission, with more substantial evidence in the pediatric population. Avoiding foods that may exacerbate or complicate symptoms is recommended, and reintroducing foods should be gradual. Some patients, especially those with obstructive complications, require intestinal surgery, and preoperative treatment with enteral nutrition can improve nutritional status [11]

**Figure 2:**
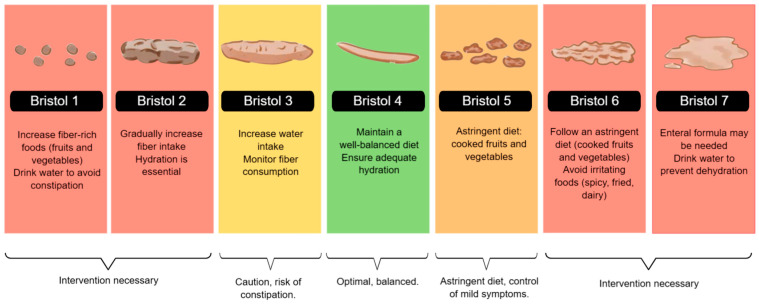
Bristol Stool Chart and Nutritional Recommendations. The figure illustrates the Bristol Stool Scale and corresponding dietary recommendations. It guides fiber intake, hydration, and enteral nutrition strategies to manage stool consistency and optimize gastrointestinal function.

In Figure 2, we illustrated dietary recommendations based on stool consistency, using a color-coded system aligned with the Bristol Stool Scale. Patients with hard stools (Bristol types 1 and 2) may benefit from increasing fiber and fluid intake, while those with softer stools (types 5 and 6) should follow an astringent diet to control symptoms. Patients with severe diarrhea (Bristol 7) may require enteral nutrition or intensive hydration strategies to prevent dehydration [11,47,48].

## 6. Non-Recommended Diets and Their Impact on IBD Progression

In contrast to therapeutic dietary interventions, specific moderate eating patterns have been consistently associated with adverse clinical outcomes and increased disease activity in patients with IBD. These nutritional patterns include ultra-processed foods and the Western diet.

### 6.1. Ultra-Processed Foods

Among the many risks associated with the development of IBD, a diet high in ultra-processed foods (UPF) has been linked to the type of ingredients they contain, such as non-caloric sweeteners (NCS), additives, artificial flavors, added sugars, stabilizers, emulsifiers, and preservatives, which can damage the intestinal barrier, trigger an inflammatory cascade, and alter the patient’s immune response [49,50]. These compounds can lead to bacterial translocation and increase bacterial adherence to the intestinal epithelium in the presence of *Escherichia coli*, potentially causing bacterial overgrowth and more significant bacterial infiltration into the intestinal villi. Additionally, the consumption of UPF has been associated with the development of diseases like colon cancer, cardiovascular diseases, and a higher incidence of IBD [50]. Specifically, NCS, which are currently consumed in large amounts in the Western diet as an alternative to sweet foods, have been negatively associated with intestinal homeostasis and increased gastrointestinal symptoms, including diarrhea, postprandial discomfort, constipation, and other gastrointestinal disorders [49,51]. Moreover, food additives present in UPF may promote bacterial virulence, increase pathogenic bacteria growth, decrease beneficial bacteria growth, and increase gut permeability. In IBD, evidence suggests that UPF may be harmful, primarily due to its dysbiosis of the gut microbiota [52].

### 6.2. Western Diet

The Western diet is a contemporary eating pattern characterized by the consumption of large amounts of UPF, refined grains, red and processed meats, NCS, sugary beverages, sweets, fried items, conventionally raised animal products, high-fat dairy products, and foods high in fructose [53]. In IBD patients, there is a growing body of research recommending reducing the intake of red meat, processed meat, and saturated fats. Specifically, red meat has been associated with a higher risk of developing IBD. A study comparing a group of people with occasional red meat consumption to another group with frequent consumption found that the likelihood of developing IBD was higher in the group with higher intake, and it was also associated with more relapses and increased disease activity [54]. Furthermore, an imbalance in the consumption of omega-3 and omega-6 fatty acids has been linked to increased IBD activity. It has been reported that a higher intake of omega-6 fatty acids is associated with a greater risk of developing UC. In contrast, high consumption of omega-3 fatty acids and docosahexaenoic acids (DHA) may reduce the risk of developing UC. However, the imbalance in the consumption of these two fatty acids in the diet is considered a risk for the development of IBD [55]. Western food is becoming more available in the developing world. In different reviews of the global epidemiology of IBD, it was noted that there has been an increase in the incidence and prevalence of IBD in parts of Asia and Africa, particularly in urban areas and in higher socio-economic classes. These changes have been associated with a reduction in gut microbial diversity, which is a feature of IBD [56]. Consumption of a Western diet may reduce the density and function of small intestinal Paneth cells, a type of epithelial cell with innate immune function, potentially contributing to the pathogenesis of IBD [57].

**Figure 3:**
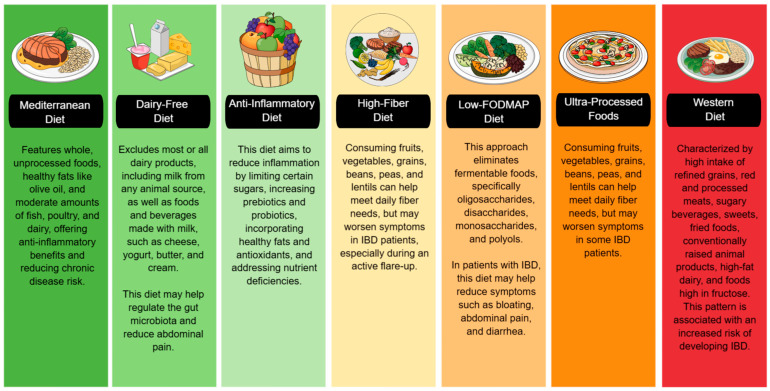
Using a color-coded system, this figure categorizes diets based on their impact on IBD. The green section includes diets that promote gut health and reduce inflammation. The yellow and orange sections indicate diets that can help reduce bloating and diarrhea but may also be potentially triggering. The red section warns against increased inflammation and an increased risk of IBD. IBD: Inflammatory Bowel Disease, FODMAP: Fermentable Oligo-, Di-, Monosaccharides, and Polyols.

The impact of various diets on IBD is increasingly recognized, with dietary patterns influencing inflammation, gut microbiota, and disease activity. While no single diet is ideal for all IBD patients, some may be more appropriate in specific clinical situations. For example, a low-FODMAP diet may be helpful in patients with predominant gastrointestinal symptoms such as bloating, pain, or diarrhea. In contrast, a high-fiber diet may benefit those without active inflammation by helping to modulate the gut microbiota. Strategies such as an anti-inflammatory or dairy-free diet may help reduce inflammation and alleviate symptoms during flare-ups. Due to its holistic, anti-inflammatory, and evidence-backed approach, the Mediterranean diet is often considered one of the most comprehensive long-term diets. However, combining elements from different diets—such as the high nutrient density of the Mediterranean diet, the restriction of FODMAP irritants, and the inclusion of functional foods from the anti-inflammatory diet—may be a more effective strategy, provided it is tailored to the patient’s characteristics, digestive tolerance, and clinical status. However, it is essential to consider that the greater the number of dietary restrictions, the greater the risk of compromising nutritional status and unnecessarily prolonging treatment. Therefore, a balance between therapeutic efficacy and maintaining nutritional status must always be sought (Figure 3).

## 7. Nutritional Support in IBD

Nutritional support is often necessary, and in some cases, it is the most effective treatment strategy, especially in patients with significant weight loss, intestinal surgery, bowel obstruction, or severe inflammation, which hinders adequate nutrient intake. In some cases, the use of enteral nutrition (EN) or parenteral nutrition (PN) is essential to maintain the nutritional status of patients and reduce complications [58].

Nutritional support should be initiated when oral intake is insufficient (<50% of energy requirements for more than 7 days), in cases of preoperative malnutrition, short bowel syndrome, high-output fistulas, or severe malabsorption [58].

### 7.1. Enteral Nutrition

It is as effective as corticosteroids at inducing remission in 73% of pediatric patients on an intention-to-treat basis, but not in adults. It is used when the digestive tract is functional, but oral intake is insufficient. It can be exclusive or supplemental, particularly in active disease states. In the perioperative context, preoperative EN has been shown to reduce the risk of infections and postoperative complications significantly. Among patients who received EN, complications were 21.9%, compared to 73.2% of those without EN. The gastrointestinal tract must be functional for EN to effectively ensure nutrient absorption. It is recommended for patients receiving steroids, as it provides benefits in protein turnover without negatively affecting disease activity [19].

EN can be administered via nasogastric tube or percutaneous endoscopic gastrostomy, depending on the patient’s tolerance and duration of treatment. An enteral feeding pump is preferred over bolus administration, mainly if a jejunal tube is used [19].

A standard polymeric formula with moderate fat content is recommended for formula selection. Specific supplements, such as glutamine or omega-3 fatty acids, are not advised, as there is no clear evidence of their benefit. No significant differences exist between using elemental, semi-elemental, and polymeric formulas in inducing remission in CD. In patients with bile salt malabsorption, medium-chain triglyceride (MCT) formulas may be a suitable option [19,59].

There are two primary methods: nasoenteric and ostomosis, and both require a formula for 6–8 weeks. The formula used can be isotonic (300–350 mOsm/kg), polymeric, and hyperproteic (1.2–1.5 g/kg/day), with a standard caloric content of 25–30 kcal/kg/day. Standard regimens may be used where refeeding syndrome is a risk (with lower calories) or where catch-up nutrition is required—a long-term method in which a direct opening is created to the digestive tract. If tolerable, oral nutrition is resumed.

The most common complications of EN include mechanical complications, such as tube obstruction or malposition; metabolic complications, including electrolyte imbalances or hyperglycemia; and infectious complications related to tube contamination or infections at the gastrostomy site. The use of PN should only be considered when EN is not feasible or is contraindicated. Conditions such as complete bowel obstruction, paralytic ileus, severe shock, intestinal ischemia, high-output fistula, or severe gastrointestinal bleeding prevent the use of EN and necessitate PN [19].

### 7.2. Parenteral Nutrition

PN is most frequently used on CD with severe complications, while in UC, it is only indicated in select cases. PN is recommended when EN is not possible, such as in cases of short bowel syndrome, high-output fistulas, intestinal obstruction, gastrointestinal bleeding, or severe digestive dysfunction [19].

It is used when the digestive tract is non-functional due to stenosis, intestinal fistulas, gastrointestinal bleeding, or obstruction [60].

Depending on the duration of treatment and the osmolarity of the administered solution, parenteral nutrition (PN) can be administered peripherally or centrally. Peripheral Parenteral Nutrition (PPN) is administered through a peripheral vein, allowing for the rapid initiation of nutritional support without needing a central access device. However, PPN has significant limitations, as its osmolarity must be restricted to less than 900 mOsm/L in pediatrics and 800–850 mOsm/L in adults to prevent phlebitis and thrombophlebitis. Additionally, the low reliability of peripheral access limits its use to no more than 10–14 days, and the need for diluted solutions reduces its ability to meet nutritional requirements fully. For these reasons, PPN is typically used as partial supportive therapy or as a bridge to Central Parenteral Nutrition (CPN) in patients requiring central access but without immediate availability [61].

When PN is required for more than 10–14 days, Central Parenteral Nutrition (CPN) is recommended, as it allows the infusion of hyperosmolar solutions (>800–850 mOsm/L) without the risk of peripheral venous damage. For administration, the catheter must be placed in the superior vena cava or right atrium, using venous access points such as the subclavian, internal jugular, cephalic, or basilic veins, while avoiding femoral vein access whenever possible due to its higher risk of infection and thrombosis [58,61].

**Figure 4:**
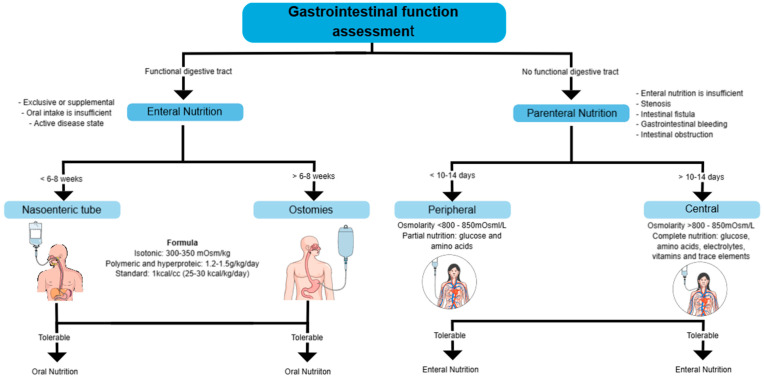
Assessment of gastrointestinal function and selection of nutritional support. This diagram outlines the decision-making process for determining the appropriate nutritional support based on gastrointestinal function.

The choice between PPN and CPN should be based on the anticipated duration of nutritional support, the patient’s clinical condition, and the feasibility of transitioning to EN, ensuring appropriate management to prevent metabolic and infectious complications (Figure 4) [58,60,61].

## 8. Psychological Impact of Disease and Nutrition in Patients with IBD

Recently, studies have reported that the gastrointestinal and extra-intestinal symptoms that patients may have affect their quality of life. These patients may have a higher prevalence of psychopathologies such as anxiety and depression compared to healthy individuals [62]. Anxiety and depression in patients with IBD have been reported to be 29% to 35% during periods of remission and 60% to 80% during active periods of the disease [63].

The most frequent symptoms associated with stress are fecal incontinence and a feeling of urgency, as well as anticipatory anxiety about having episodes in public. This is followed by pain, fatigue, fear of a new attack, sleep quality, and dietary restrictions, which are often a cause of distress and anxiety [62]. In an analysis based on questionnaires administered to patients with IBD, it was found that a significant proportion of patients had unhealthy eating habits, associated with symptoms such as fatigue, nervousness, and a greater tendency toward stress and emotional eating. This suggests that an inadequate diet, combined with a lack of rest or exercise, negatively contributes to the psychological well-being of patients with IBD [64].

It has been widely studied that a better quality diet, characterized by a higher consumption of fruits, vegetables, omega-3 fatty acids, zinc, magnesium, and selenium, is associated with lower anxiety levels [65]. These recommendations are common in Mediterranean dietary patterns. It has been shown that nutrition can improve mental health; for example, combining a Mediterranean diet and mindfulness practices has been associated with better health-related quality of life. This dietary pattern, rich in fruits, vegetables, healthy fats, and antioxidants, could reduce systemic inflammation, improve the gut microbiota, and modulate the gut–brain axis, which would positively affect both the digestive and psychological levels [66]. Regarding the relationship between daily anxiety and dietary restriction, momentary anxiety and dietary restriction have been observed to be positively and significantly associated, both within and among individuals. In a study using an ecological momentary assessment protocol, participants reported that when they experienced more anxiety than usual, they tended to try to restrict their eating [67].

On the other hand, recent studies have shown that poor sleep quality, along with elevated levels of anxiety and depression, are significantly associated with greater clinical disease activity [68]. Therefore, promoting a comprehensive nutritional approach that considers the reduction of digestive symptoms and the improvement of the patient’s emotional state is essential in managing IBD. Further research is needed into nutritional interventions that integrate psycho-emotional and behavioral components as part of interdisciplinary treatment.

## 9. Conclusions

Nutritional assessment is a fundamental component in the treatment of patients with IBD. A multidisciplinary approach, including a comprehensive dietary assessment throughout the disease, can prevent vitamin and mineral deficiencies and alterations in body composition, improving quality of life. The dietary pattern followed by patients, along with the types of foods they exclude or consume most frequently, can influence disease courses, clinical activity, gastrointestinal symptoms, changes in body composition, and alterations in biochemical values.

Dietary therapies are emerging as a promising tool in managing IBD, with evidence supporting diets such as the Mediterranean, dairy-free, anti-inflammatory, and high-fiber diets as effective strategies for reducing inflammation, improving symptoms, and supporting remission in patients with this disease. However, patient response to different therapies can vary, and personalizing the diet based on individual needs and characteristics is crucial. Although available studies indicate benefits in various aspects of IBD, further research is needed to elucidate the precise mechanisms and determine the long-term impact of these dietary interventions.

Patients with IBD are at high risk of malnutrition, so implementing systematic nutritional assessments is essential to identify and prevent deficiencies. Dietary management for these patients is diverse, incorporating various nutritional strategies, some of which have more substantial scientific evidence than others. Adjusting the diet based on stool consistency is an effective strategy for managing acute symptoms. Nutritional support is necessary for patients who cannot meet their caloric or protein requirements through oral intake alone, with EN as the first-line therapy.

## 10. Limitations of the Included Studies

Despite the relevant findings regarding the influence of diet on IBD, it is important to note that several studies reviewed present some limitations that should be considered when interpreting the results. Some investigations are observational, which limits the ability to establish causal relationships. In addition, several studies relied on self-reported data collection methods, which may introduce recall and social desirability biases. Heterogeneity was also observed in the criteria used to assess dietary adherence and in the differences in follow-up periods.

## Abbreviations

The following abbreviations are used in this manuscript:

IBD	Inflammatory Bowel Disease
CD	Crohn’s Disease
UC	Ulcerative Colitis
EN	Enteral Nutrition
PN	Parenteral Nutrition
NPP	Peripheral Parenteral Nutrition
NPC	Central Parenteral Nutrition
MASLD	Metabolic dysfunction-associated steatotic liver disease
SCFAs	Short-Chain Fatty Acids
CRP	C-reactive protein
FODMAP	Fermentable Oligo, Di-, Monosaccharides and polyols
UPF	Ultra-processed foods
TCM	Medium-chain triglycerides
